# Why Humans Fail in Solving the Monty Hall Dilemma: A Systematic Review

**DOI:** 10.5334/pb.274

**Published:** 2018-06-01

**Authors:** Lore Saenen, Mieke Heyvaert, Wim Van Dooren, Walter Schaeken, Patrick Onghena

**Affiliations:** 1 Laboratory for Experimental Psychology, KU Leuven, Leuven, BE; 2 Educational Effectiveness and Evaluation, KU Leuven, Leuven, BE; 3 Instructional Psychology and Technology, KU Leuven, Leuven, BE; 4 Methodology of Educational Sciences, KU Leuven, Leuven, BE

**Keywords:** Systematic review, Monty Hall dilemma, probability, choice, decision

## Abstract

The Monty Hall dilemma (MHD) is a difficult brain teaser. We present a systematic review of literature published between January 2000 and February 2018 addressing why humans systematically fail to react optimally to the MHD or fail to understand it.

Based on a sequential analysis of the phases in the MHD, we first review causes in each of these phases that may prohibit humans to react optimally and to fully understand the problem. Next, we address the question whether humans’ performance, in terms of choice behaviour and (probability) understanding, can be improved. Finally, we discuss individual differences related to people’s suboptimal performance.

This review provides novel insights by means of its holistic approach of the MHD: At each phase, there are reasons to expect that people respond suboptimally. Given that the occurrence of only one cause is sufficient, it is not surprising that suboptimal responses are so widespread and people rarely understand the MHD.

## Introduction

The notorious Monty Hall dilemma (MHD) was adapted from the popular TV game show *Let’s Make a Deal* (Friedman, 1998). The problem is also known as the ‘Three Doors Problem’ and is mathematically equivalent to the ‘Three Prisoners Problem’ (e.g., Shimojo & Ichikawa, 1989). In the classic version of the MHD, a guest is confronted with three identical doors. One door conceals a valuable prize, usually a car. The two remaining doors conceal worthless prizes, such as goats. After the guest makes an initial choice for one door, the host, who is aware of the location of the prize, opens a non-chosen door to show that there is a worthless prize behind it. Next, the guest is asked whether he wants to stay with his initial choice, or wants to switch to the remaining unopened door.

By applying Bayes’ Theorem with the correct prior, and marginal likelihoods, it can be derived that switching is the optimal behaviour with a probability of 2/3 to win the prize, while sticking to the initial choice only yields a 1/3 probability. The rationale for these posterior probabilities can be explained by looking at Table 1. With three identical doors concealing one car (i.e., the prize) and two goats (i.e., worthless or mock prizes) randomly placed behind the doors, there are three possible sequences: The car might be hidden behind door A, door B, or door C. Suppose that a contestant initially chooses door A. Table 1 shows that staying with the initial choice (i.e., door A) will lead to a winning ratio of 1/3 (see sequence 1), whereas switching will lead to a winning ratio of 2/3 (see sequences 2 and 3). Note that in sequence 1, the host can open either door B or door C, whereas in sequences 2 and 3, the host has no other choice than opening the door revealing the *other* goat (i.e., door C and door B respectively). The same ratios emerge when the contestant would make an initial choice for door B or door C. Thus, the optimal solution to the MHD is to switch.

**Table 1:** Possible sequences, choices and outcomes in the MHD.

Sequence	Door A	Door B	Door C	Initial choice		Initial choice		Initial choice	
				Door A		Door B		Door C	

				Final choice		Final choice		Final choice	

				Stay	Switch	Stay	Switch	Stay	Switch

1	**Car**	**Goat**	**Goat**	Win	Lose	Lose	Win	Lose	Win
2	**Goat**	**Car**	**Goat**	Lose	Win	Win	Lose	Lose	Win
3	**Goat**	**Goat**	**Car**	Lose	Win	Lose	Win	Win	Lose

Total number of wins:				1	2	1	2	1	2

However, the vast majority of people shows a strong tendency to stick with their initial choice (Burns & Wieth, 2004; Friedman, 1998; Granberg, 1999a; Granberg & Brown, 1995; Granberg & Dorr, 1998). Cross-cultural research revealed that sticking percentages range between 79% and 87% (Granberg, 1999a). Also when people solve repeated trials of the MHD, sticking percentages remain relatively high (e.g., Granberg & Dorr, 1998). Besides this suboptimal behaviour, most people have the strong idea that their choice, either staying or switching, does not matter, because they consider the probability to win the prize for both options as being equal (Franco-Watkins, Derks, & Dougherty, 2003; Granberg & Brown, 1995; Stibel, Dror, & Ben-Zeev, 2009). Note that both people’s equiprobability reasoning and sticking behaviour are not in line with the optimal solution to the MHD.

The MHD is a valuable research topic precisely because of its highly counterintuitive solution and the difficulties that people experience to understand it. People hold several misconceptions about (posterior) probabilities (e.g., Batanero & Sanchez, 2005; Garfield & Ahlgren, 1988; Shaughnessy, 1992) of which some play an important role in the MHD as well (e.g., equiprobability bias). In their review, Tubau, Aguilar-Lleyda, and Johnson (2015) pointed to similitudes and differences between the MHD and other Bayesian problems. They stated that “differing from most Bayesian problems, prior and conditional probabilities in the MHD have to be inferred” (p. 1). The aim of the current review is to identify all known causes for people’s suboptimal performance on the MHD for which at least some empirical evidence is found. By providing such review, we aim to complement the more theoretical review of Tubau et al. (2015) who compared the MHD to other Bayesian tasks.

The current paper thus provides a *systematic* literature review addressing the overarching question why humans are so bad in solving and understanding the MHD, being one of the most counterintuitive probability problems. The review provides a structured overview of all known erroneous reasoning processes and misconceptions people have about the MHD and which thus may – but do not necessarily (see Tubau, Aguilar-Lleyda, & Johnson, 2015) – play a role in all type of areas in which posterior probabilities are involved (e.g., medical decision making). This is important for the field of statistical education as well, because “the success of any probability curriculum for developing students’ probabilistic reasoning depends greatly on teachers’ understanding of probability as well as *a much deeper understanding of issues such as students’ misconceptions* [emphasis added]” (Stohl, 2005, p. 351).

In this paper, we address three more specific research questions. First, we address the question why humans fail to solve the MHD; this may imply (a) not switching when having the choice to do so, (b) not (consistently) switching even after several experiences with the MHD, and (c) not understanding that switching doubles the chance of winning. Second, we discuss the question whether humans’ performance on the MHD can be improved. Third, we discuss the question which individual differences are related to humans’ failure of solving the MHD optimally.

## Methods

To identify, synthesize, and interpret the available research relevant to answer the posed research questions of this paper, a systematic literature review was conducted.

The article retrieving process was based on a step-by-step approach as described by Cooper (2010). Relevant articles were identified through both systematic searches of four electronic databases and hand searches of three scientific journals known to publish empirical studies considering the MHD. Subsequently, the bibliographies of the included articles were systematically examined for references to additional relevant articles. Finally, a citation index search was conducted in order to retrieve relevant articles referring to the articles already included based on the previous search steps.

Articles describing empirical, quantitative studies about the MHD that focused on a potential explanation for why humans fail to solve the MHD optimally, and/or that searched for a way to improve humans’ MHD performance were included in the systematic review. Only peer-reviewed articles in which quantitative outcomes were reported were included. Articles dealing with other aspects of the MHD, such as the logical or mathematical structure behind the problem, were excluded (e.g., Cross, 2000; de Cooman & Zaffalon, 2004). Articles which did not have the MHD as primary focus were also excluded (e.g., Patt, Bolwes, & Cash, 2006; Siddiqi, 2009). Only studies with human participants were included. Because of our interest in the most recent research regarding the MHD, only articles published in the period January 2000 – February 2018 were included. In March 2018, we conducted the most recent update of the systematic search process, for articles published up to February 2018. Articles that were published online in that period, but not yet published in a printed format, were also included if these articles could be retrieved in full text format. Only articles written in English were included.

First, the electronic searches of the databases *Embase, Eric, Pubmed*, and *Web of Science* involved the following search string: *Monty Hall* OR *Three Prisoners Problem* OR *Three Doors Problem*. This search string was used with both single and double quotation marks. One hundred and four articles were retrieved by this first search, 26 of these articles met the above specified inclusion criteria. Second, a manual screening of the journals *Thinking & Reasoning, Journal of Behavioral Decision Making*, and *Journal of Experimental Psychology: General* was performed. Based on screening titles and abstracts, 21 *additional* possibly relevant articles on the MHD were retrieved by this second search. However, after reading the 21 full text articles, we decided that none of them met all inclusion criteria. Third, the bibliographies of the included articles so far were systematically screened. No additional articles were retrieved that met the inclusion criteria. Fourth, looking for more recent research articles, a citation index search was conducted through the *Web of Science* indexes to identify articles referring to the already included articles. However, no extra articles were found that met the inclusion criteria. As a result, the final database consisted of 26 articles. The 26 included articles are marked (*) in the reference list.

The second author independently checked the retrieved articles for inclusion. Intercoder agreement was calculated by dividing the number of agreements on inclusion and exclusion by the number of agreements plus disagreements, which resulted in a percentage agreement of 98.04%. Disagreements between the first and the second author were resolved through discussion.

Data were extracted from the studies included in the review to answer the posed research questions and key characteristics of the studies were tabulated (Tables 2 and 3). The data analysis and synthesis were conducted separately for the three research questions. The data analysis occurred in close collaboration with the third author.

**Table 2:** Characteristics of included studies: participants, version, material, number of options, and number of trials.

Article	Study number	Participants	Version	Material		Number of options	Number of trials
				Alternatives	Prize (when specified)		

Baratgin & Politzer, 2010	1	47 students	Physical	Tubes	Cotton ball	3	1
	2	100 students	Physical	Tubes	Cotton ball	3	1
Burns & Wieth, 2003	/	124 students	Paper	Boxers/Doors		3	1
Burns & Wieth, 2004	1	326 students	Paper	Boxers/Wrestlers/Doors		3	1
	2	379 students	Paper	Tennis players/Boxes		3/32/128	1
	3	614 students	Paper	Boxers/Doors		3	1
	4	280 students	Paper	Doors		3	1
De Neys, 2005	1b	41 students	Computerized	Doors	Car	3	1
De Neys & Verschueren, 2006	1	239 students	Computerized	Doors	Car	3	1
	2	129 students	Computerized	Cups	Marble	3	1
	3	102 students	Computerized	Cups	Marble	3	1
DiBattista, 2011	/	189 students	- Training: computerized - Post-test: paper	Doors	Car	- Training: 3/20 - Test item: 6	- Training: unlimited - Test item: 1
Efendic & Drace, 2015	/	36 students	Computerized	Doors		3	60
Fox & Levav, 2004	1a	104 students	Physical	Cards	Ace	5	1
	1b	126 students	Physical	Cards	Ace	5	1
Franco-Watkins et al., 2003	1	142 students	- Training: physical - Final MHD: ?	- Training: cards - Final MHD: doors	- Training: ace - Final MHD: car	- Training: 3/10 - Final MHD: 3/10	- Training: 30 - Final MHD: 1
	2	259 students	- Training: computerized - Final MHD: ?	- Training: cards - Final MHD: doors	- Training: ace - Final MHD: car	- Training: 3/10 - Final MHD: 3/10	- Training: 30 - Final MHD: 1
	3	165 students	?	Doors	TV/DVD player/desk	3/4/5	3
Herbranson & Schroeder, 2010	3	12 students	Computerized	Squares		3	200
Herbranson & Wang, 2014	2	12 students	Computerized	Squares		3	100
Hirao et al., 2016	/	32 students	Computerized	Cards	Money	3	120 (2 × 60) in each condition
Hirao et al., 2017	/	21 students	Computerized	Cards	Money	3	360 (6 × 60)
Howard et al., 2007	/	108 students	Computerized	Boxes	Money	3 → 25	23
Idson et al., 2004	/	95 students	Combination of paper and computerized	Boxes		3	1 MAO, 1 MM
Klein et al., 2013	/	15 students	Computerized	Black squares	Green square	3	500 or end of 60 minutes session (whichever came first)
Krauss & Wang, 2003	1	135 students	?	Doors	Car	3	1
	2	137 students	?	Doors	Car	3	1
	3	110 students	?	Doors/Prisoners	Car/Prisoner not executed	4/3	Each problem once
Mazur & Kahlbaugh, 2012	2	36 students	Computerized	Boxes		3	200
	3	16 preschoolers (ages 3–5 years)	Computerized	Doors	Fun picture	3	50
Petrocelli, 2013	/	10 physicians	Computerized	Doors	Money	3	60
Petrocelli & Harris, 2011	1	47 students	Computerized	Doors	Money	3	60
	2	65 students	Computerized	Doors	Money	3	60
Saenen et al., 2015a	/	385 students	Physical	Cups	Toy	3/10/50	10
Saenen et al., 2015b	/	68 students	Physical	Cards	Red card	3	80
Slembeck & Tyran, 2004	/	93 students	Computerized	Doors		3	40
Stibel et al., 2009	1	32 students	?	Boxes	Money	3/100	1
	2	152 students	?	Boxes	Money	5/6/7/8/9/10	1
	3	82 students	Computerized (online survey on internet)	Boxes	Money	3	1
	4	61 students	?	Boxes	Money	3/100 (both with removal of initial choice)	1
Tubau, 2008	1a	44 students	?	Cards	Ace	3	1
	1b	40 students	?	Cards	Ace	3	1
	2	40 students	?	Cards	Ace	3	1
	3	32 students	?	Cards	Ace	3	1
Tubau & Alonso, 2003	1	57 students	Computerized	Windows	Red window	3	45
	2	61 students	Physical	Cards	Ace	3	18
	3	60 students	Paper	Cards/Envelopes	Ace/Money	3	1

*Note.* Question marks (?) indicate that the information could not be retrieved from the article.

**Table 3:** Characteristics of included studies: design, independent and dependent variable.

Article	Study number	Design	Independent variable(s)	Dependent variable(s)

Baratgin & Politzer, 2010	1	2 × between	Instructions	Probability judgment (winning when staying)
	2	2 × within	Instructions	Probability judgment (winning when staying)
Burns & Wieth, 2003	/	2 × between	Problem context	- Behaviour: switching/staying - Probability judgment (winning when switching) in frequencies
Burns & Wieth, 2004	1	4 × between	Problem context	- Behaviour: switching/staying - Probability judgment (winning when switching)
	2	3 × 2 between	- Number of options - Problem context	- Behaviour: switching/staying
	3	2 × between	Problem context	- Behaviour: switching/staying - Probability judgment (winning when switching and staying) in frequencies
	4	2 × between	Training	- Behaviour: switching/staying - Probability judgment (winning when switching and staying) in frequencies
De Neys, 2005	1b	2 × between: results 1b compared to results 1a	Working memory load	Behaviour: switching/staying/equal
De Neys & Verschueren, 2006	1	Correlational	Working memory capacity	Behaviour: switching/staying/equal
	2	Correlational	Working memory capacity	Behaviour: switching/staying/equal
	3	2 × between	Working memory load	Behaviour: switching/staying/equal
DiBattista, 2011	/	No systematic manipulation		- Behaviour: switching/staying - Correct explanation
Efendic & Drace, 2015	/	2 × between	Instructions	- Behaviour: switching/staying
Fox & Levav, 2004	1a	3 × between	Behaviour of host	- Behaviour: trading or not - Probability judgment
	1b	2 × between	Behaviour of host	- Behaviour: trading or not - Probability judgment
Franco-Watkins et al., 2003	1	2 × 2 × 2 between	- Choice restriction - Number of options during training - Number of options in final MHD	- Behaviour training: switching/staying - Behaviour final MHD: switching/staying - Probability judgment
	2	2 × 2 × 2 × 2 between	- Number of options during training - Number of options in final MHD - Probability reinforcement for switching - Hypothetical player’s decision	- Behaviour final MHD: switching/staying - Probability judgment
	3	- 3 × between - 3 × within	- Number of options - Prize	- Behaviour: switching/staying - Probability judgment (winning when switching and staying)
Herbranson & Schroeder, 2010	3	2 × between	Probability reinforcement for switching	Behaviour: switching/staying
Herbranson & Wang, 2014	2	2 × between	Distribution of unequal base rates across the options	- Behaviour: initial choice - Behaviour: switching/staying
Hirao et al., 2016	/	3 × within	Probability reinforcement for switching	- Behaviour: switching/staying - Probability judgement (winning when switching and staying) - Physiology: stimulus-preceding negativity (SPN)
Hirao et al., 2017	/	No systematic manipulation		- Behaviour: switching/staying - Probability judgement (winning when switching and staying) - Physiology: stimulus-preceding negativity (SPN)
Howard et al., 2007	/	4 × between	- Representation of second stage information - Number of options	Behaviour: switching/staying
Idson et al., 2004	/	2 × between	Training type	Behaviour: switching/staying
Klein et al., 2013	/	2 × between: results humans compared to monkeys’ results	Species	Behaviour: switching/staying
Krauss & Wang, 2003	1	3 × between	Inclusion of four psychological elements (i.e., perspective change, one-door scenario, mental models, frequency simulation)	Behaviour: switching/staying (Classification of switchers in ‘correct explanations’, ‘correct intuition’, ‘random switchers’)
	2	4 × between	Inclusion of four psychological elements (i.e., perspective change, one-door scenario, mental models, frequency simulation)	Behaviour: switching/staying (Classification of switchers in ‘correct explanations’, ‘correct intuition’, ‘random switchers’)
	3	4 × between	Training type	Correct solution with mathematically correct justifications
Mazur & Kahlbaugh, 2012	2	3 × between	Probability reinforcement for switching	Behaviour: switching/staying
	3	Only assigned to 67% condition: results compared to students’ results	Age group	Behaviour: switching/staying
Petrocelli, 2013	/	No systematic manipulation		Behaviour: switching/staying
Petrocelli & Harris, 2011	1	No systematic manipulation		- Behaviour: switching/staying - Counterfactual thoughts following losses - Memory for decision/outcome frequencies
	2	2 × between	Counterfactual salience	- Behaviour: switching/staying - Memory for decision/outcome frequencies
Saenen et al., 2015a	/	3 × 3 between Correlational	- Age - Number of options Inhibition capacity	- Behaviour: switching/staying - Probability judgment (winning when switching or staying)
Saenen et al., 2015b	/	2 × between	- Numerical feedback format - Conditional feedback format	- Behaviour: switching/staying - Probability judgment (winning when switching or staying)
Slembeck & Tyran, 2004	/	2 × 2 between	- Communication - Competition	Behaviour: switching/staying
Stibel et al., 2009	1	2 × between	Number of options	- Behaviour: switching/staying - Probability judgment
	2	6 × between	Number of options	- Behaviour: switching/staying
	3	2 × between	Memory task	- Behaviour: switching/staying - Probability judgment
	4	2 × between	Number of options	- Behaviour: switching/staying - Probability judgment
Tubau, 2008	1 (a + b)	2 × 2 between	- Numerical format - Inclusion of a graphical representation	- Behaviour: switching/staying/equal - Probability judgment (winning when switching and staying)
	2	Correlational	Mathematical skills	- Behaviour: switching/staying/equal - Probability judgment (winning when switching and staying)
	3	Correlational	- Mathematical skills - Anagram solving skills	- Behaviour: switching/staying/equal - Probability judgment (winning when switching and staying)
Tubau & Alonso, 2003	1	No systematic manipulation		- Behaviour: switching/staying/equal - Probability judgment (winning when switching and staying) in frequencies
	2	3 × between	- Participant’s role - Perspective effect	Behaviour: switching/staying
	3	3 × between	- Perspective effect - Explicit representation	- Behaviour: switching/staying/equal - Probability judgment (winning when switching and staying)

**Table 4:** Overview of the different phases in the MHD, its related causes explaining humans’ systematic failure on the MHD, and its related interventions to improve humans’ MHD performance.

Research question	Phase in the MHD			
	Phase 1: Before elimination	Phase 2: After elimination	Phase 3: After one completed trial	Phase 4: After multiple trials

Research question 1: Causes explaining humans’ suboptimal MHD performance	- Difficulties with initial probability understanding - Illusion of control	- Failure to take into account the host’s dependent behaviour - Partitioning-editing-counting - Updating - Anticipated regret/General reliance on affect - Conservative response tendency (stimulus-preceding negativity)	- Counterfactual thinking - Experienced regret	- Probability matching - Distorted memory for decision/outcome frequencies
Research question 2: Improving humans’ MHD performance	- Numerical representation - Number of options	- Emphasis on the underlying structure - Representation of conditional information		- Experience due to multiple trials - Conditional frequency feedback - Probability reinforcement - Competition (and communication)

Table 2 provides an overview of various characteristics of the included studies. First, the number of participants for each study is mentioned. Second, it is distinguished whether the study was computerized, a paper-and-pencil task, or performed as a physical task (e.g., by using cards). Third, an inventory of the used materials used to operationalize the alternatives (i.e., doors in the classic MHD) and the prize (i.e., a car in the classic MHD) is made. Fourth, the numbers of options are mentioned, demonstrating that most studies included three alternatives analogous to the classic MHD. A limited number of studies increased the number of options significantly, up to 100 (Stibel et al., 2009) and 128 (Burns & Wieth, 2004) alternatives. Finally, the number of MHD trials are mentioned, showing that most studies investigated just one MHD trial, whereas other studies let participants solve repeated MHD trials.

Table 3 provides an overview of the study design, the independent variable(s), and the dependent variable(s) of all included studies. As can be derived, the majority of studies used a between-subjects design. Next, a very large variation in the independent variables can be observed. On the other hand, the dependent variables used were rather homogeneous across studies, mostly focusing on the choice behaviour and/or probability judgements of participants. Two recent studies, however, also focused on the brain activity of participants while solving the MHD (Hirao, Murphy, & Masaki, 2016, 2017).

The current review focuses on humans’ MHD performance. The term ‘performance’ is used to refer to MHD performance in general, and thus may refer to choice behaviour and/or (probability) understanding of the MHD. The term ‘behavioural performance’ is only used to refer to participants’ choice behaviour (either staying or switching). The term ‘understanding’ is mostly used to refer to probability judgments, in accordance with how ‘understanding’ is usually operationalized in the research literature (see Table 3). An exception to this operationalization of ‘understanding’ is found in the research of DiBattista (2011), who investigated whether participants could “correctly explain” the MHD solution. It is important to be aware that ‘understanding’ can only be inferred from overt responses, such as correct probability judgements. It has to be noted, however, that people may realize that the posterior probabilities for staying and switching are 1/3 and 2/3 respectively, without understanding the *cause* of this advantage.

## Results

In order to answer the three research questions of this paper, the following structure will be used. First, we give an overview of possible causes that contribute to the answer on the question why humans do not give optimal switching responses to the MHD when having the choice to do so – even after previous experience with the problem – and do not understand that switching is beneficial. This section will be structured using a sequential analysis of the MHD, in which we discuss various causes that may occur at each step and that prevent humans from solving the MHD optimally. Second, we will discuss empirical studies that searched for how performance on the MHD can be improved, based on the same sequential analysis that was introduced earlier. Also training studies that were conducted to improve MHD performance will be discussed. Third, individual differences that are related to humans’ failure to solve the MHD optimally will be discussed.

### Possible Causes Explaining Humans’ Suboptimal MHD Performance

In this section, we will provide an overview of the causes that explain humans’ failure to solve the MHD (on both the behavioural and the understanding level) as reported in the literature. A close reading of the articles included in this systematic review revealed that different causes play a role at different phases of the MHD. Therefore, a sequential analysis of the MHD will be used to structure those causes. More specifically, we will discuss four phases in the MHD in which different problems may occur, all prohibiting humans to solve the MHD in an optimal way and to fully understand the problem. An overview of the different phases in the MHD and its related causes can be found in Table 4.

**Phase 1: Before Elimination.** The first phase in the MHD is the situation *before* a door is eliminated by the host. Here, the contestant is confronted with three identical choice alternatives and is asked to pick one of the three doors, knowing that only one door conceals the prize. The literature indicates two problems may arise at this point, leading a participant towards a suboptimal MHD performance.

First, research of Tubau (2008) showed that participants can have difficulties understanding the initial probabilities in the MHD. Furthermore, the results showed that the ability to express the initial probabilities is a good predictor of solving the dilemma correctly (Tubau, 2008). Evidently, it would be very difficult for a participant to detect the optimal behaviour and the underlying posterior winning probabilities without having a good understanding of the initial probabilities.

Second, in order to explain the research results demonstrating that after solving repeated trials, switching rates were significantly higher for pigeons compared to humans (Herbranson & Schroeder, 2010), Herbranson (2012) discussed that humans might be influenced by the illusion of control in this phase of the MHD. Although the initial choice is inconsequential in the classic MHD, with no influence over the chance of winning, participants do in fact make an overt initial choice, one that they might very well interpret as meaningful. Participants might for example, avoid switching because they believe that their initial choice is the most likely to be the winner. In short, difficulties in the MHD can occur even *before* the actual dilemma is posed.

**Phase 2: After Elimination.** The second phase in the MHD is the situation *after* the host eliminated a door. The contestant is now confronted with two remaining choice alternatives and is asked to either stay with his initial choice, or to switch to the other remaining alternative. The literature review indicates that at this point, there are five causes explaining why humans fail to solve the MHD in an optimal way.

First, people fail to notice or to keep in mind the crucial information that the host is aware of the location of the prize and that he will never open the door containing the prize. Thus, humans sometimes fail to take into account the knowledgeable behaviour of the host. To understand the MHD, one should realize that the correct door is initially chosen in one third of the cases, and that only in those cases the host will randomly open one of the two remaining non-winning doors (cf. Table 1). However, in two thirds of the cases the contestant will initially pick the wrong door and therefore, the host does not have a choice: There is only one non-winning door left that is not initially picked by the contestant (cf. Table 1). Participants do not seem to realize that the behaviour of the host is dependent on their own behaviour. In several studies, attempts were made to make this dependent behaviour of the host more explicit, as will be discussed below (Burns & Wieth, 2003, 2004; Idson et al., 2004; Krauss & Wang, 2003; Tubau & Alonso, 2003). Note that if the host’s behaviour were not constrained (i.e., the host can open the door containing the prize), there is no advantage to switching (Herbanson & Schroeder, 2010; Idson et al., 2004).

Second, Fox and Levav (2004) and Franco-Watkins et al. (2003) investigated posterior probability judgments after participants solved (variants of) the MHD. Their results demonstrated that in order to calculate the posterior probabilities, participants typically divide the number of prizes by the number of remaining options after conditional information is provided. This method to calculate probabilities is called the partition-edit-count strategy (Fox & Levav, 2004) and is consistent with the equiprobability bias (Lecoutre, 1992): The winning probabilities for staying and switching are judged to be equal, because the location of the prize is considered to be completely random. Whereas partitioning-editing-counting (Fox & Levav, 2004) is a specific strategy to estimate probabilities, the literature also identifies specific types of probability revision, such as ‘updating’ and ‘focusing’ (Baratgin & Politzer, 2010).

Updating is the third cause we discuss in this section, explaining why people may fail to solve the MHD. The difference between updating and focusing can be explained as follows (see Baratgin & Politzer, 2010). When focusing, probability revision takes place in a stable situation. Specifically for the MHD, the initial situation of three doors does still exist, but when one door is removed by the host, a participant focuses on the two remaining alternatives. When updating, probability revision takes place as if the situation has evolved. In other words, participants revise their probabilities after new information is provided as if the situation has changed from the initial situation. Specifically in the MHD, the situation initially consists of three doors. However, after the host provides information about the door that does not contain the prize, people update this situation: The new situation only involves two doors and previous information about the non-winning door is no longer considered relevant. Baratgin and Politzer (2010) found evidence for updating when people solve the MHD. In their experiment, participants were asked to give posterior probability estimates of winning when staying. They overwhelmingly judged this probability to be 1/2 instead of 1/3. Note that these results are consistent with the partition-edit-count strategy as described by Fox and Levav (2004). However, it should be emphasized that updating may – but *does not necessarily* – lead participants to believe that posterior winning probabilities for staying and switching are equal. Hence, updating can also be *in*consistent with the equiprobability bias. For example, a participant can update the probabilities based on the erroneous belief that after the elimination of one option by the host, the probability is higher for his initial choice than for the remaining one. This way of updating the MHD situation can explain people’s documented tendency to stay with the initial choice, although as far as we know, this hypothetical explanation for people’s sticking tendency has never been investigated. Finally, it has to be mentioned that partitioning-editing-counting (Fox & Levav, 2004) can be applied as well when focusing, and thus can be applied either when updating or focusing (Baratgin & Politzer, 2010).

Fourth, people’s suboptimal MHD performance can be explained by the larger amount of regret participants anticipate to experience after a loss due to switching rather than to a loss due to staying (Stibel et al., 2009), or by a more general reliance on affect (Efendic & Drace, 2015). In Stibel et al.’s (2009) experiment, participants solved one trial of (a variant of) the classical MHD. For one group of participants, the influence of regret was eliminated by removal of the first choice: A computer randomly picked a door being the first choice. Participants’ switching responses were higher compared with those of participants who themselves made the initial choice. In the study of Efendic & Drace (2015), participants were assigned either to the control condition or to the reliance on affect condition. Participants of the latter condition repeatedly received the instruction to take into account how positive or negative they felt regarding their choice behaviour and to rely on their affect and emotions in order to make their final choice. Participants assigned to the reliance on affect condition switched statistically significantly less compared to participants in the control condition. Although (anticipated) regret or a more general reliance on affect cannot explain people’s lack of understanding the MHD, it *can* explain why people prefer to stay with their initial choice, despite the fact that they judge the winning probabilities for switching and staying as equal.

Fifth, Hirao, Murphy and Masaki (2016) investigated the brain activity of participants confronted with the MHD. They explained that people do not switch when having the choice to do so by the fact that humans have a strong conservative response tendency. In their research, larger stimulus-preceding negativity was found when participants stayed with their initial choice compared to switching behaviour (Hirao et al., 2016). The stimulus-preceding negativity is an event-related potential associated with the affective-motivational anticipation of feedback in gambling tasks and develops between an action and the outcome of a decision task. The authors discuss that larger stimulus-preceding negativity may be associated with the illusion of control and/or with the anticipation of regret. It has to be noted, however, that in the study of Hirao et al. (2016), participants who frequently changed their initial choice, were excluded from the ERP analysis.

**Phase 3: After One Completed Trial.** The third phase in the MHD is the situation in which the contestant has received feedback about the outcome of his final decision, in the sense that he won or lost the trial depending on his final choice for a door by either staying or switching.

In the literature, it is argued that at this point, people may engage in counterfactual thinking (Petrocelli, 2013; Petrocelli & Harris, 2011). Counterfactual thinking is defined as thinking about an alternative decision that could have been made or thinking about different outcomes of a decision that has been made (e.g., Roese, 1997). People are especially prone to counterfactual thinking after experiencing a negative outcome (e.g., Boninger, Gleicher, & Strathman, 1994) or after having chosen to do something rather than having decided to do nothing (e.g., Gilovich, Medvec, & Chen, 1995; Landman, 1987).

Petrocelli and Harris (2011) found evidence that participants produced more counterfactuals after switch losses (e.g., “If I had stayed, I would have won”) than after stay losses, and that counterfactuals after switch losses prescribe dysfunctional responses in that they motivate the participants to stay with their initial choice in *subsequent* trials. Note that the role of regret might be interwoven with a biased counterfactual thinking: People regret switch losses more than stay losses (Stibel et al., 2009) and besides this, people produce more counterfactuals after switch losses than after stay losses (Petrocelli & Harris, 2011).

**Phase 4: After Multiple Trials.** The last phase in the MHD only takes place when a participant is confronted with a series of completed MHD trials, in which he experiences the outcome of his choices several times. At this point, the literature identifies two causes explaining why humans fail to solve the MHD.

First, research has shown that people show signs of probability matching (Herbranson & Schroeder, 2010). In the experiment of Herbranson and Schroeder (2010), participants solved multiple trials of the MHD. Due to the experience of repeated trials, participants were able to estimate the probability of winning when switching, and subsequently matched their behaviour by switching in approximately the same proportion of cases as their estimated probability of winning when switching. This line of research indicates an adaptation of behaviour based on reinforcement, but does not necessarily refer to any rational understanding of the problem. On the contrary, a rational understanding of the problem would imply that one switches on all trials in order to maximize the likelihood of winning. Note that participants who demonstrate this behaviour could be functioning under the assumption that there is a way to be accurate on all trials. If so, then probability matching might reflect their futile search for perfect accuracy.

Second, the experiments of Petrocelli and Harris (2011), already mentioned above, showed that people do not correctly monitor the success and loss outcomes for staying and switching. More specifically, participants were asked to estimate their win and loss frequencies conditional on the performed behaviour (i.e., staying or switching) after completing multiple trials of the MHD. Results showed that after repeated trials, people’s memory for decision/outcome frequencies is typically distorted: Participants overwhelmingly overestimated the switch losses they had experienced during the experiment (Petrocelli & Harris, 2011).

All included studies of this systematic review in which participants received feedback, either conditional or non-conditional, show that participants fail to fully understand the advantage of switching because 100% switching behaviour was never observed (DiBattista, 2011; Herbranson & Schroeder, 2010; Hirao et al., 2016, 2017; Klein, Evans, Schultz, & Beran, 2013; Mazur & Kahlbaugh, 2012; Saenen, Van Dooren, & Onghena, 2015b; Slembeck & Tyran, 2004).

### The Improvement of Humans’ MHD Performance

Below, we will discuss the retrieved empirical studies on how humans’ MHD performance could be improved. They will be presented in accordance with the sequential analysis of the MHD that was provided above, which demonstrated that different phases of the MHD are related to specific causes prohibiting humans to solve the MHD optimally and to understand the problem. Table 4 provides an overview of the different phases in the MHD and its related manners in order to improve humans’ performance.

Furthermore, we will discuss training studies that demonstrated how MHD performance could be improved.

**Phase 1: Before Elimination.** So far, we already showed that people may have problems with the MHD even *before* the actual dilemma emerges. The research literature demonstrates two manipulations at this stage of the problem which have shown to improve people’s MHD performance.

First, Tubau (2008) showed that the numerical representation of the MHD may be a critical factor for the performance on this task, at least for participants with lower mathematical skills (see above). According to the author, the numerical presentation of the MHD is important to investigate because previous research on posterior probabilities indicated the positive effect of presenting posterior probability problems in natural frequencies compared with probabilities (e.g., Gigerenzer, 1991, 1994). In her research, two groups of participants were confronted with the MHD. Participants who were stimulated to think in terms of natural frequencies switched more often compared to participants who were encouraged to reason in terms of probabilities (Tubau, 2008).

Second, several studies manipulated the number of options (i.e., more doors) in the MHD (Burns & Wieth, 2004; Franco-Watkins et al., 2003; Saenen et al., 2015a; Stibel et al., 2009). A participant’s intuition that the initial choice is the correct one will be less probable with an increased number of options, because the difference between the prior probabilities to initially choose the correct versus wrong option becomes larger. In a 100-door variant, for instance, the probability to initially pick the winning door is only 1/100 and the probability to initially pick a wrong door is 99/100, whereas in a 3-door variant, those probabilities equal 1/3 and 2/3 respectively. Results of the three retrieved studies that manipulated the number of options showed that switching behaviour was more likely to occur when more options were included in the MHD (Burns & Wieth, 2004; Franco-Watkins et al., 2003; Saenen et al., 2015a; Stibel et al., 2009). Saenen et al. (2015a) and Stibel et al. (2009) also investigated the posterior probability judgments of participants. The great majority of participants judged the posterior winning probabilities as .50, which indicates a dissociation between behavioural MHD performance and understanding the underlying probabilities. Results of the experiment of Saenen et al. (2015a) showed that with a higher number of options (i.e., 10 and 50), however, participants gave statistically significantly more often correct posterior probability judgements for both staying and switching compared to participants who solved the classic MHD with three options. Important to notice is that Stibel et al. (2009) explain the effect of higher switching rates by the influence of working memory capacity, claiming that memory capacity becomes overloaded when the number of options in the MHD increases. Therefore, they conclude that lower working memory resources result in better MHD performance. Note that this conclusion is inconsistent with research of De Neys (2005) and De Neys and Verschueren (2006) described above. However, in our opinion, the experiments in which Stibel et al. (2009) investigated the manipulation of the number of options do not reflect an investigation of the influence of working memory capacity on MHD performance. We do not agree that for example a 100-door MHD necessarily requires more working memory capacity than a 3-door MHD. Rather, better MHD performance when more options are included can be explained by the intuition that the participant’s initial choice will probably (not) be the correct one, which we assume to be less available with an increased number of options.

**Phase 2: After Elimination.** Several studies investigated how manipulating the presentation of the actual dilemma influenced human’s MHD performance, as we will describe below.

A problem that may arise at this stage of the MHD is the failure to take into account the fact that the behaviour of the host is dependent on the initial choice made by the contestant (see above). In order to overcome this neglect, several authors conducted studies in order to emphasize that the behaviour of the host is not random, and that it instead conveys important information (Burns & Wieth, 2003, 2004; Idson et al., 2004; Krauss & Wang, 2003; Tubau & Alonso, 2003).

Idson et al. (2004) gave participants two versions of the MHD in paper-and-pencil format: “Monty Always Opens” (MAO) and “Mean Monty” (MM). The MAO refers to the classical MHD, in which the host *always* opens a non-winning door, not being the door initially chosen by the participant. The MM refers to the situation where the host *might* decide to open a non-winning door, not being the door initially picked by the participant: When the participant initially picked the correct door, Monty would open a non-winning door; when the participant initially picked the wrong door, Monty would never open a door. Thus, in the MM problem, the optimal solution for a participant would be to always stick with his initial choice, which would lead to a prize in one third of the cases (while switching – when possible – would lead to never winning the prize). Participants were assigned to an implicit or explicit training condition. Participants in the implicit training condition received both MHD problems sequentially. In the explicit training condition, participants received both MHD problems displayed side-by-side. Participants were asked whether in order to have the best chance of winning the prize, one should switch. For the MAO, the correct response is “yes”, whereas for the MM problem, the correct response is “no”. More correct answers were observed for participants assigned to the explicit training condition compared to participants assigned to the implicit training condition. The authors therefore concluded that comparative and analogical processing helps to improve MHD performance. In our opinion, presenting both problems side-by-side, the intentional behaviour of Monty was made more explicit.

Another way to make the behaviour of the host more explicit was investigated by Tubau and Alonso (2003). They conducted an experiment in which the MHD was played between two adversaries (one participant was assigned to the role of the host, and the other participant was the contestant). A significant improvement in switching responses was observed compared to performance in the classic version of the MHD, which the authors see as a perspective effect. This effect holds that participants, who play against each other, build mental models from the perspectives of *both* players. In line with the study of Tubau and Alonso (2003) are the results of Krauss and Wang (2003). In their study, perspective change was induced by asking participants to imagine they were the host of the game show. According to the authors, by taking the perspective of the host, his intentional behaviour will become more obvious for the participant. This perspective change manipulation (at least in combination with a problem stated in natural frequencies) led to a higher switching rate (Krauss & Wang, 2003).

Related to these findings are the results of Burns and Wieth (2003, 2004), who tried to make the underlying structure of the MHD more explicit by making an analogy with a boxing competition context. In this context, the contestant placed a bet on one of three boxers, then the two un-chosen boxers did a fight, and finally the contestant could change from his initially chosen boxer to the winner of the fight. The authors argue that this analogy would reveal the causal structure underlying the MHD: Two autonomous factors (the contestant’s choice and the host’s choice) influence a sole outcome. Results showed that putting the MHD in such context indeed led to higher switching responses compared with responses to the classical MHD.

Next, Howard, Lambdin, and Datteri (2007) showed the positive effect of highlighting the conditional nature of the chances involved in the MHD, given that one of the three doors is opened. The authors manipulated the visual access and representation of the non-winning item(s), assuming that making the second-stage information (such as in the classical MHD in which a non-winning door is opened) more salient would facilitate switching behaviour, especially if this second-stage information would be made more explicit by increasing the size of the non-winning item(s). Participants switched most often in the condition in which the non-winning un-chosen box received the label “empty” (i.e., “empty” condition), and least often in the condition in which the non-winning un-chosen box was removed from the screen (i.e., “vanish” condition). Thus, in the empty condition the non-winning un-chosen box was visualized by appearance of the word “empty” on it, whereas in the vanish condition this box totally disappeared. Note that the latter condition suggests the emergence of a new situation in which the conditional information is considered to be irrelevant, which may facilitate engagement in updating (see Baratgin & Politzer, 2010). Participants that were assigned to a condition in which the non-winning un-chosen box was removed from the screen and either the initially chosen box or the initially non-chosen box was made larger, scored better than participants in the “vanish” condition, but worse than participants in the “empty” condition.

**Phase 3: After One Completed Trial.** Obviously, manipulations occurring after an MHD trial is completed cannot influence the performance on that trial. This immediately explains why our systematic review did not reveal any studies for this subsection.

**Phase 4: After Multiple Trials.** Explanatory factors for humans’ suboptimal MHD performance after multiple trials are probability matching and a distorted memory for decision/outcome frequencies. Multiple studies in which participants had to complete a series of trials showed an improvement of MHD performance. These studies will be discussed in this section.

First, in several studies, participants were given multiple trials of the MHD which allowed the researchers to investigate whether repeated experience and feedback with the problem would improve performances (Efendic & Drace, 2015; Franco-Watkins et al., 2003; Herbranson & Schroeder, 2010; Herbranson & Wang, 2014; Hirao et al., 2016, 2017; Klein et al., 2013; Mazur & Kahlbaugh, 2012; Petrocelli, 2013; Petrocelli & Harris, 2011; Saenen et al., 2015b; Slembeck & Tyran, 2004; Tubau & Alsonso, 2003). The results of most of these studies provide strong evidence for learning from experience: Increased switching rates over trials were observed (Franco-Watkins et al., 2003; Herbranson & Schroeder, 2010; Hirao et al., 2017, Klein et al., 2013; Mazur & Kahlbaugh, 2012; Petrocelli, 2013; Petrocelli & Harris, 2011; Saenen et al., 2015b; Slembeck & Tyran, 2004; Tubau & Alonso, 2003). However, in none of these studies the repeated experience led to participants consistently switching on all trials. Thus, despite repeated experience, participants failed to learn the absolute and unqualified advantage of switching. In addition, in the study of Herbranson and Wang (2014) and in one experimental condition (with the classical MHD) of the study of Mazur and Kalhbaugh (2012), participants showed no significant increase in switching rates over trials. These participants, however, already showed a high switching rate in the first block of trials as compared to other studies (Franco-Watkins et al., 2003; Klein et al., 2013; Petrocelli, 2013; Petrocelli & Harris, 2011; Saenen et al., 2015b; Slembeck & Tyran, 2004). An important question that arises with the evidence of increased switching behaviour across successive trials is whether participants gain understanding of the problem as well. From the above mentioned studies, Franco-Watkins et al. (2003), Hirao et al. (2016, 2017), Saenen, Van Dooren, and Onghena (2015b), and Tubau and Alonso (2003) asked participants to judge their posterior winning probabilities. Results showed that despite of increased switching rates, correct probability judgments were rarely mentioned. Therefore, there exists a clear dissociation between behavioural performance on and (probability) understanding of the MHD.

An alternative explanation for increased switching rates without increased understanding is the phenomenon of probability matching that was explained above. Unfortunately, several articles do not report the observed switching percentages (e.g., exact switching percentages and standard errors, as well as individual switching profiles) in sufficient detail, so it is impossible to investigate whether the improvement in switching rates is merely due to a probability matching trend or not (e.g., Franco-Watkins et al., 2003; Klein et al., 2013; Petrocelli, 2013; Petrocelli & Harris, 2011). Several other articles *do* report results that in principle could be explained by the probability matching phenomenon (e.g., Efendic & Drace, 2015; Herbranson & Schroeder, 2010; Herbranson & Wang, 2014; Hirao et al., 2017; Mazur & Kahlbaugh, 2012). However, an analysis of individual profiles (as reported in Herbranson & Wang, 2014) would still be required in order to find more direct evidence for probability matching in specific participants.

Second, Hirao et al. (2017) and Saenen et al. (2015b) investigated the effect of feedback on participants’ MHD performance. In the experiment of Saenen et al. (2015b), participants were assigned to one of four conditions created by a 2 × 2 between subjects design: Feedback was provided either in frequency or in percentage format, and was either conditional or non-conditional. Each participant completed a series of 80 MHD trials. Updated feedback about the performance was provided after each trial. Results showed that switching rates increased most when feedback was provided in conditional frequency format. However, correct posterior probability judgments were rarely given; thus, the permanent feedback did not help participants to understand why switching was beneficial (see above). In the study of Hirao et al. (2017), participants completed 360 MHD trials constantly receiving feedback in conditional frequency format. Meanwhile, their stimulus-preceding negativity was measured. Different from Hirao et al. (2016), this study focused on the brain activity of participants who, due to conditional frequency feedback and repeated trials, learned to adapt the optimal behaviour. The results showed that stimulus-preceding negativity over frontal regions deceased in the second half of the experiment compared to the first half. Throughout the entire experiment, larger stimulus-preceding negativity was found on switch trials compared to staying behaviour (Hirao et al., 2017).

Third, several studies investigated the influence of probability reinforcement for switching. The idea behind these studies is that in the classical MHD, the difference in frequency of winning by switching vs. staying (2/3 vs. 1/3) is not sufficiently salient for participants, especially in a limited number of trials. The influence of probability reinforcement of switching was investigated in experiments of Franco-Watkins et al. (2003), Herbranson and Schroeder (2010), Hirao et al. (2016), and Mazur and Kahlbaugh (2012). In their experiments, participants received a series of trials of the classical three-door MHD, but the probability reinforcement for switching was manipulated between conditions, going from 10% to 90%. Notice that such manipulation can only be done in computerized experiments. In all four studies, higher switching rates were observed in conditions with higher probability reinforcements (Franco-Watkins et al., 2003; Herbranson & Schroeder, 2010; Hirao et al., 2016; Mazur & Kahlbaugh, 2012). Thus, with greater differences between the probability of reinforcement for staying and switching, participants seemed to notice more easily the advantage of switching. However, probability matching might be an alternative explanation for these results.

Fourth, Herbranson and Wang (2014) investigated the effect of manipulating the base rates of the available options. The base rates of the three available options were .40, .30, and .30 in the one condition, and .20, .40, and .40 in the other condition. In order to optimally solve an MHD variant with unequal base rates, one should initially select the option (or one of the options) that has the lowest probability of containing the prize and subsequently switch to the other remaining option. Thus, the optimal solution here involves two consecutive behaviours and is more complex to pick up compared to the optimal behaviour of merely switching in the classic MHD. In both conditions, however, participants failed to apply the optimal behaviour. When only investigating participants’ switching rates, no increase across trials was observed, which may have to do with the relatively high switching rate participants showed in the first block of trials (see above).

Fifth, Slembeck and Tyran (2004) investigated the influence of communication and competition on MHD performance. Participants solved the MHD 40 times and were assigned to one of four conditions, created by a 2 × 2 between subjects design. The first independent variable was communication. Participants either solved the MHD individually or in small groups of three subjects in which they were allowed to communicate with each other. The second independent variable was competition. Participants were rewarded with money depending on either their own performance, or their performance relative to that of other subjects (or groups of subjects). The highest switch rates were observed in the condition where participants were both allowed to discuss the MHD with each other (communication) and where the reward for their performance was relative to the performance of others (competition). Thus, the combination of communication and competition led to an increase of switching responses. Competition is clearly a factor that belongs to the fourth phase of the MHD, because participants were rewarded based on their performance after all 40 trials had been completed. It should be noted, however, that communication belongs to all four phases defined in the MHD, because communication was allowed during the entire experiment. Because of practical reasons (i.e., reporting the entire study at once), the effect of communication is discussed here together with the effect of competition.

**Training Studies.** Above, we discussed studies that investigated how alterations in a specific phase of the MHD could improve humans’ performance on the task. There also exist several studies that investigated the effect of different types of training on humans’ MHD performance. Their aim was to investigate which type of interventions with particular characteristics (whether systematically manipulated or not) would improve behavioural performance on and/or (probability) understanding of the MHD (Burns & Wieth, 2004; DiBattista, 2011; Franco-Watkins et al., 2003; Krauss & Wang, 2003).

In an experiment by Burns and Wieth (2004), participants either received training tasks on the underlying structure of the MHD or no training. Participants in the training condition received problems with an analogue structure (e.g., in a boxing competition and musician context) to the MHD and were provided with explanations about why switching was the optimal behaviour. At the end of the study, all participants completed a standard version of the MHD. Participants assigned to the training condition revealed significantly more switching responses on the MHD and could more often give the correct answer to the posterior probability question.

Next, Krauss and Wang (2003) conducted an experiment in which the participants first had to complete the standard version of the MHD (pre-test). None of the participants was able to give the correct mathematical solution to the problem. Next, participants were assigned to either a training condition or a control condition. Afterwards, participants were asked to solve both the three prisoners problem and a four-door version of the MHD (post-test). When participants received a training where the MHD solution was explained in terms of natural frequencies, or a training on mental models, they performed better on the two post-test problems.

DiBattista (2011) investigated whether training with an interactive digital learning object would improve performances on a variant of the MHD. In his study, participants solved the classical MHD in a paper-and-pencil format as a pre-test measure. Next, they were motivated to practice both a 3-door and a 20-door version of the MHD using a digital interactive learning object. They could access the learning object as many times as they wanted. Several elements of the learning object were specifically designed in order to increase MHD performance: Constantly updated feedback about the number of times participants won or lost the game, separately for staying and switching, and the provision of a cumulative summary of outcomes in both percentages and frequencies. Furthermore, participants could access explanations about the underlying structure of the MHD (i.e., an explanation about the intentional behaviour of the host). After five weeks of practicing with the learning object, participants performed very well on a six-door variant of the MHD: They often chose the correct behaviour (switching) and could often give a satisfactory explanation for this solution. Which task features of the learning object were responsible for this performance however cannot be confirmed, because none of the specifically designed elements to improve performances were experimentally manipulated between (or within) participants. Four weeks after the six-door variant MHD testing, DiBattista (2011) included a post-test for the classical MHD. According to us, this post-test has no value, as participants could literally reproduce the correct behaviour and correct explanation from their experience with the digital learning object.

Also Franco-Watkins et al. (2003) investigated the effect of training. In their research, the similarity of 30 repeated trials of the MHD and a final trial of the MHD was manipulated. Participants solved either 30 consecutive 3-card MHD problems or 30 consecutive 10-card MHD problems. After those 30 trials, participants either solved a final 3-door or 10-door MHD. Results showed that following the 10-card game, a greater number of participants switched in the final 10-door MHD than in the final 3-door MHD, whereas following the 3-card game similar switching levels were observed for the final 3-door and 10-door MHD. The authors also examined probability judgments made by the participants. Despite influenced switching behaviour, the similarity between the card game and the final MHD problem did not facilitate the generation of correct posterior probability judgments. As Saenen et al. (2015b), Tubau and Alonso (2003), and Stibel et al. (2009), Franco-Watkins et al. (2003) concluded that there exists a dissociation between implicit knowledge obtained from the game (increased switching behaviour) and the explicit understanding why switching is the optimal behaviour in the MHD (increased winning probabilities for switching). In a second experiment, Franco-Watkins et al. (2003) manipulated the similarity of 30 repeated 3-card or 10-card MHD trials solved by a hypothetical player and a final trial of the 3-door or 10-door MHD solved by the participant. Results were consistent with their previous findings: Congruent situations (3-cards game & 3-door MHD, or 10-cards game & 10-door MHD) produced slightly more switching responses than the incongruent situations (3-cards game & 10-door MHD, or 10-cards game & 3-door MHD). Correct posterior probability judgments were still rarely observed.

Summarized, these training studies show that when participants were trained on the underlying structure of the MHD (Burns & Wieth, 2004; Krauss & Wang, 2003), frequency formulation (Krauss & Wang, 2003), received congruent training tasks (Franco-Watkins et al., 2003), or received training on a combination of factors (DiBattista, 2011), their behavioural MHD performance increased. Sometimes even their posterior probability judgments were correct after receiving training (Burns & Wieth, 2004; DiBattista, 2011; Krauss & Wang, 2003).

### Individual Differences Related to Humans’ Suboptimal MHD Performance

When searching for explanations why humans fail to solve the MHD optimally, a question that arises is whether there are individual differences between persons that may provide an explanation. In what follows, we will discuss research in which the influence of general individual differences on MHD performance was investigated. Note that these individual differences cannot be discussed specific to the phases of the MHD as used in the previous paragraph, because it is (still) unclear whether and how those subject characteristics influence human’s MHD performance in each specific phase.

First, Mazur and Kahlbaugh (2012) and Saenen et al. (2015a) investigated whether participants of various age groups differ in their MHD performances. In the study of Mazur and Kahlbaugh (2015), participants were either preschoolers (ages 3–5 years) or adult students. Results showed no statistically significant different switching rates between both groups. In the study of Saenen et al. (2015a), participants were either primary school students (norm ages: 10 and 11 years), secondary school students (norm ages: 14 and 15 years), or university students (norm age: 18 years). When solving the first MHD trial, no effect of age was found. When solving 10 MHD trials, however, an interaction effect between age group and the number of options (i.e., 3, 10, or 50) did predict behavioural responses. In the 3-doors MHD, switching behaviour decreasing with increasing age, whereas in the 10-doors MHD, the opposite effect was demonstrated. In the 50-doors MHD, no effect of age group was found, which was explained by a ceiling effect. After having experienced 10 trials, participants completed a questionnaire in which they were asked to indicate the optimal behaviour in order to maximize winning probabilities and to judge posterior probabilities. Both secondary school students and university students more often correctly indicated switching as the optimal behaviour. They also more often correctly judged the posterior probability for staying compared to primary school students. Interestingly, no age effect was found for the posterior probability judgement of switching.

Second, Saenen et al. (2015a) demonstrated that a higher ability to inhibit intuitive erroneous answers positively correlated with better MHD performances: Participants who solved all three items on the MHD questionnaire correctly (i.e., optimal behavioural response and correct posterior probability judgments for both staying and switching – defined as “full MHD understanding” by the authors) performed statistically significantly better on an inhibition task compared to participants who did not show this “full MHD understanding”.

Third, a person’s mathematical skills are related to MHD performance. Tubau (2008) showed that for participants with high mathematical skills, the numerical presentation of the MHD does not matter. For participants with lower mathematical skills, however, the numerical presentation of the MHD was important, as they reasoned correctly more often when the MHD was stated in natural frequencies compared to probabilities.

Fourth, De Neys and Verschueren (2006) found that a higher working memory capacity was correlated with higher switch rates. A correlation however does not imply that working memory is causally related to MHD performances. Therefore, De Neys (2005) and De Neys and Verschueren (2006) manipulated working memory resources while participants completed an MHD trial. Results showed that when working memory capacity was experimentally burdened by higher secondary task load, participants were less likely to switch. This leads to the conclusion that higher working memory capacity may facilitate optimal behaviour. However, an experiment of Stibel et al. (2009) led to contrary results. Participants solved the MHD while performing a classic memory task. Results indicated that more switching responses occurred when working memory resources were burdened by the memory task. Note however, that the inconsistent results may be due to the method used by Stibel et al. (2009), who used an internet-based survey, and a working memory burdening task that was not actively conducted *while* solving the MHD.

## Discussion

An advantage of systematic reviews is that a comprehensive, explicit, and reproducible data collection process is followed. The systematic review process allows the identification of key findings, of reasons for different results across studies, and of limitations of current knowledge. Our systematic literature review about the MHD was performed in order to address the question why humans systematically fail in solving the MHD. This overarching question was translated in three more specific research questions: (1) Which factors explain humans’ failure to solve the MHD (which may imply (a) not switching when having the choice to do so, (b) not (consistently) switching even after several experiences with the MHD, and (c) not understanding that switching doubles the chance of winning and thus is beneficial); (2) Can humans’ MHD performance be improved; and (3) Which individual differences are related to humans’ failure of solving the MHD optimally?

Considering the first research question, our systematic review revealed that humans’ failure to solve the MHD can be explained by an analysis of the sequential structure in the MHD. More specifically, causes leading participants towards a suboptimal MHD performance can occur at four different phases. First, before the elimination and thus even before the dilemma occurs, humans may have difficulties with understanding the initial probabilities (Tubau, 2008) or they may be influenced by the illusion of control (Herbranson, 2012). Second, after elimination and thus when the contestant is confronted with the actual dilemma, humans may fail to take into account the dependency of the host’s behaviour (Burns & Wieth, 2003, 2004; Idson et al., 2004; Krauss & Wang, 2003; Tubau & Alonso, 2003). Furthermore, partitioning-editing-counting (Fox & Levav, 2004; Franco-Watkins et al., 2003), updating (Baratgin & Politzer, 2010), and the anticipation on regret (Stibel et al., 2009) or a more general reliance on affect (Efendic & Drace, 2015) may prevent humans from arriving at the optimal solution. Also humans’ conservative response tendency may play a role (Hirao et al., 2016). Third, after one MHD trial is completed, counterfactual thinking (Petrocelli & Harris, 2011) and experienced regret (Stibel et al., 2009) may prevent humans from arriving at a full understanding of the MHD and may strengthen suboptimal MHD performances on subsequent trials. Fourth, after experience with multiple MHD trials, humans may engage in probability matching (Herbranson & Schroeder, 2010) and/or may have a distorted memory for decision/outcome frequencies (Petrocelli & Harris, 2011), again leading to suboptimal MHD performances.

Summarized, in different phases of the MHD, specific problems can occur explaining why humans fail to solve the MHD. We argue that the proposed sequential analysis of the MHD and its related causes provides a comprehensive and clear overview of the evidence available in the literature; no other previous study approached the explanations for suboptimal performance in the MHD in such a way. One of the possible implications of this sequential analysis is that humans’ systematic failure to fully understand and optimally solve the MHD can now be more easily understood: At each phase, there are reasons to expect persons to give a suboptimal response, and given that the occurrence of only one cause is already sufficient to prevent a person to respond optimally on the MHD, it is not surprising that suboptimal responses are so widespread. Note that in all four phases, there are causes that can explain why participants do not switch when having the choice to do (cf. research question 1a) and why they do not understand that switching doubles the chance of winning (cf. research question 1c). The causes belonging to the fourth phase (i.e., probability matching and a distorted memory for decision/outcome frequencies) specifically can explain why participants do not (consistently) switch after having experienced multiple MHD trials (cf. research question 1b).

Considering the second research question, our systematic review revealed that authors sometimes did not clearly state which rationale underpinned their research. The answer, however, could most frequently be found in the sequential analysis we proposed. For example, Burns and Wieth (2003, 2004), Idson et al. (2004), Krauss and Wang (2003), and Tubau and Alonso (2003) all investigated whether making the dependency of the host’s behaviour more explicit would improve participants’ MHD performances.

The results of most studies that aimed to improve humans’ MHD performance showed that humans’ behavioural performance could be relatively easily improved by particular manipulations. However, optimal performance (i.e., consistent switching behaviour) was typically not observed. One reason may be that although participants’ *behavioural performance* on the MHD was improved, there usually were no indications that they also gained *understanding* of the problem. In many studies, only behavioural performance (in terms of switching rates) was considered, but studies that did take into account (probability) understanding as well (e.g., Franco-Watkins et al., 2003; Hirao et al., 2016, 2017; Saenen et al., 2015a, 2015b; Stibel et al., 2009; Tubau & Alonso, 2003) clearly demonstrated that there exists a dissociation between behavioural MHD performance and understanding (the underlying probabilities of) the problem. Accordingly, people may feel, experience, or create an intuition that switching has an advantage over staying, but they will presumably not know *why* this is the case. We believe this an important finding that warrants further research. As can be seen in Table 3, most studies only focused on behavioural MHD performance (De Neys, 2005; De Neys & Verschueren, 2006; DiBattista, 2011; Efendic & Drace, 2015; Herbranson & Schroeder, 2010; Herbranson & Wang, 2014; Howard et al., 2007; Idson et al., 2004; Klein et al., 2013; Mazur & Kahlbaugh, 2012; Petrocelli, 2013; Petrocelli & Harris, 2011; Slembeck & Tyran, 2004) and there is one article that only addressed participants’ probability judgments (Baratgin & Politzer, 2010). We encourage all researchers to include both behavioural responses and probability judgments as dependent variables in their future research (see Burns & Wieth, 2003, 2004; Fox & Levav, 2004; Franco-Watkins et al., 2003; Hirao et al., 2016, 2017; Krauss & Wang, 2003; Saenen et al., 2015a, 2015b; Stibel et al., 2009; Tubau, 2008; Tubau & Alonso, 2003).

The fact that many studies succeeded in improving participants’ behavioural MHD performance, but further failed to report results consistent with optimal behavioural responses and a full understanding of the problem, can again be explained by the sequential analysis of the MHD proposed in this paper. Because there are many explanatory factors in the MHD which (mis)lead participants towards suboptimal performance, influencing only one or some of those factors will never be sufficient in order to arrive at optimal behavioural MHD performance and a full understanding of the (underlying probabilities of the) problem. Instead, studies should focus on the entire sequential structure of the MHD and its related causes of suboptimal performance.

Considering the third research question, our systematic review revealed that a better inhibition ability, higher mathematical skills and higher working memory resources are beneficial for humans’ MHD performances. Age did sometimes influence humans’ MHD performances, depending of the study and of which dependent variables were investigated (see Saenen et al., 2015a).

Regarding the individual differences related to MHD performance, our systematic review further showed that it is not clear *why* particular individual differences are important. Although research was conducted to determine the influence of some subject characteristics on humans’ MHD performances, at this point it has not yet been investigated why a better inhibition ability, higher mathematical skills and higher working memory capacity contribute to better MHD performances.

By performing this systematic literature review about humans’ MHD performance, we were also able to detect some knowledge and research gaps. First and not surprisingly, there is still a lack of knowledge about how actual understanding of the MHD can be improved. Although some studies already include probability judgments as a dependent variable in their experiments besides the behavioural dependent variable (e.g., Burns & Wieth, 2003, 2004; Fox & Levav, 2004; Franco-Watkins et al., 2003; Hirao et al., 2016, 2017; Krauss & Wang, 2003; Saenen et al., 2015b; Stibel et al., 2009; Tubau, 2008; Tubau & Alonso, 2003), studies so far mostly failed to improve correct understanding. Future research should focus on improving humans’ understanding of the MHD, by relying on the entire sequential analysis of the problem.

Second, our literature review revealed that some factors are just not yet investigated enough. For example, the influence of age was only investigated by Mazur and Kahlbaugh (2012) and Saenen et al. (2015a). Age did not seem to influence MHD performance in the study of Mazur and Kahlbaugh (2012), but did seem to play a role in the study of Saenen et al. (2015a). These two studies (with only two and three different age groups, respectively) cannot provide enough evidence for a statement whether age does matter or not (and if so, how) in how people solve the MHD. Note that another study of De Neys (2007) – not included in this review because the research was not published in a peer reviewed journal but as a book chapter – suggests that age might actually matter: Twelve and thirteen year old students showed higher switching responses than senior high school and university students.

Finally, the relationship between individual differences and MHD performance needs more research. As stated before, so far it is only known that particular subject characteristics play a role in how the MHD is solved. However, it is unclear *why* these characteristics are important. Future research should focus on the question whether particular causes of our sequential analysis are more important for individuals with certain characteristics than for other individuals. Therefore, it might be worth it to consider single case research designs (Gerring, 2007; Kazdin, 2011) when investigating people’s MHD performance. Granberg (1999b) for example, noted that some subjects eventually adopt the optimal solution and begin switching on every trial, especially in the condition in which the difference between the probability reinforcements for staying and switching was made larger. The latter result is known from studies included in our systematic review (Franco-Watkins et al., 2003; Herbranson & Schroeder, 2010; Mazur & Kahlbaugh, 2012). We need to be aware that the large majority of MHD research so far reported results of data analyses on group level, but did not analyze participants’ *individual* data patterns. This implies that there may have been participants in other studies than the study of Granberg (1999b) who showed the same data pattern of consistently switching, but that they were not noticed because no analyses were performed on the individual level.

Although this systematic review focused on the MHD, our findings may have implications beyond the dilemma itself. Stohl (2005) pointed to the importance of teachers’ knowledge of students’ and teachers’ own misconceptions regarding (posterior) probabilities in order to develop successful probability education. Because of the strongly counterintuitive solution of the MHD, it is considered to reveal many erroneous reasoning processes and misconceptions. Indeed, our systematic review revealed various causes (updating, not taking into account conditional information, etcetera) and misconceptions (e.g., the equiprobability bias), which are known to occur in some other (posterior) probability problems (see Garfield, 2003). We need to be aware, however, that our systematic review provided a sequential analysis of the MHD specifically and its related causes explaining humans’ suboptimal MHD performance. When another (posterior) probability problem would be investigated, another overview of causes for suboptimal performance could be found (see Tubau et al., 2015).

However, given the importance of correct probability reasoning in domains such as medical decision making and Supreme Court decision making, it is crucial to understand why people fail to correctly deal with (posterior) probabilities and what can help them to understand probabilities better. The findings of our systematic review illustrate that erroneous posterior probability reasoning in the MHD can happen in various phases and can happen due to various causes. Unfortunately, no elementary intervention can overrule all those erroneous reasoning processes, or as Herbranson (2012) stated: “Avoidance of a single common bias or misunderstanding is not enough – one must dodge several in order to maximize the likelihood of winning” (p. 300). Because of the difficulty of understanding (posterior) probabilities and the persistency of some misconceptions, we advise mathematics and statistics teachers to pay attention to and educate their students about the misconceptions that may occur in both the MHD and other problems dealing with (posterior) probabilities. Awareness of the difficulty and trickiness of (posterior) probabilities can be a first step towards better (posterior) probability reasoning.
